# Improvement of a slimming cream's efficacy using a novel fabric as a transdermal drug delivery system: An ***in vivo*** and ***in vitro*** study

**DOI:** 10.3892/etm.2020.8582

**Published:** 2020-03-06

**Authors:** Kwang Ho Yoo, Tae-Rin Kwon, Chang Taek Oh, Kyeung Chan Ko, Yong Hwan No, Won Jong Oh, Beom Joon Kim

**Affiliations:** 1 Department of Dermatology, College of Medicine, Chung-Ang University Hospital, Seoul 156-755, Republic of Korea; 2 Department of Dermatology, College of Medicine, Catholic Kwandong University, International St. Mary's Hospital, Incheon 22711, Republic of Korea; 3 Research and Development Center, Green Cross Well-Being Corporation, Seongnam 13595, Republic of Korea

**Keywords:** novel fabric, slimming cream, transdermal drug delivery system

## Abstract

Penetration of any compound into the body from the outside is prevented primarily by the corneal layer of the epidermis. The only way to circumvent the properties of the corneal layer is to disrupt it. Currently, transdermal systems can currently only deliver drugs that are of low molecular weight. The purpose of the present study was to assess the improvement of the slimming cream's efficacy using a novel fabric, with the aim of developing an improved method for transdermal drug delivery. The current study was conducted on four groups of guinea pigs. The control group was untreated, whereas the test groups were treated with either slimming cream and no fabric, slimming cream with 100% cotton fabric or slimming cream with the novel fabric. Ultrasound and microscopic histological analysis were used to assess animals. The results demonstrated that compared with the other groups, the novel fabric group demonstrated the greatest reductions in fat layer thickness, adipocyte size and number and proliferator-activated receptor-γ levels in adipose tissue. Furthermore, the novel fabric also enhanced the transdermal delivery of rhodamine B base and caffeine penetration compared with the control fabric (3.18-fold). In conclusion, the results of the present study demonstrated that the novel fabric can potentially be used to enhance transdermal drug delivery.

## Introduction

Human skin has many unique properties, including its function as a physicochemical barrier. This property protects the body from dangerous pathogens however, it also complicates the delivery of therapeutic agents and resists the penetration of a number of molecules (1). Skin penetration follows the ‘500 Dalton rule’, therefore, it is difficult for hydrophilic therapeutic molecules of large molecular weight to penetrate the normal skin barrier (2). This is problematic since it is often important for dermatologists to deliver effective ingredients to a targeted layer of skin.

To overcome this difficulty, a number of methods can be used to temporarily increase the permeation of drugs through the skin barrier (3,4). These methods include chemical, biochemical and physical approaches. In particular, chemical enhancers have been developed that increase the diffusibility of the substance across the barrier, increase product solubility in the vehicle and improve the partition coefficient (5). Furthermore, the manipulation of lipid biosynthesis has allowed the modification of the barrier structure itself to increase drug penetration. However, in some cases these modifications result in skin irritations, therefore, these formulations must be carefully evaluated in a variety of preparations (6). The main physical techniques that can increase the cutaneous penetration of substances are: i) Iontophoresis, which increases the penetration of ionized substances; ii) electroporation, which electrically induces penetration through the barrier; and iii) sonophoresis, which is based on 20 to 25 KHz ultrasound and induces alterations in the skin barrier allowing the penetration of active drugs (7,8). Recently, novel physical methods, including fractional laser devices and micro-needle rollers have also been developed (9). These techniques promote drug absorption by inducing fine perforations that mildly perturb the stratum corneum (SC), thereby creating holes through which molecules can pass (10). However, the use of these modalities may result in some pain or discomfort, and can also disrupt the normal barrier function of the SC (11,12).

In contrast, a number of studies have suggested that hydration of SC lipid lamellar regions or osmotic forces in the skin can enhance the permeation of drugs through the skin (13). Water is the most natural and biocompatible penetration enhancer that has been demonstrated to improve skin permeability (14). Furthermore, recent evidence has suggested that extensive hydration using occlusion methods may disrupt lipid ultrastructure (15,16). The SC has been indicated to be a dynamic structure, in which extended hydration (>8 h) swells corneocytes, induces intercorneocyte rupture, and causes microstructural changes in lipid self-assembly (17). These disruptions allow penetration through the SC barrier. However, these disruptions are reversible, as removing the hydration source easily restores the barrier (18).

Clothing is used daily and often comes into close contact with human skin. Different types of fabric are used in clothing and effect skin moisture conditions differently and may, therefore, enable drug absorption for wound dressing, skin care and cosmetic products (19). The majority of cosmetic slimming creams contain a variety of ingredients (including caffeine, centella asiatica, ruscus, mate, retinol and *Ginko biloba*), which modulate fat storage in adipocytes (20-22).

**Figure 1:**
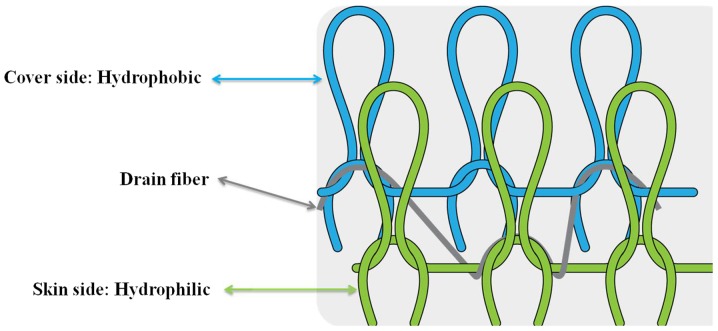
Schematic diagram of the novel drug delivery fabric.

With the aim of overcoming current limitations in transdermal delivery systems, the present study developed a novel fabric for transdermal drug delivery and evaluated its ability to enhance the effect of slimming creams. The novel fabric used in the present study consisted of two layers, an outer hydrophobic layer of polypropylene and an inner hydrophilic layer of nylon with polyurethane. This fabric creates a unique combination of conditions at the skin surface in which the hydrophobic outer layer prevents water evaporation and thus puts water in direct contact with the skin, whereas the hydrophilic layer, which is also in direct contact with the skin, maintains skin moisture (Fig. 1). Therefore, the aim of the present study was to evaluate the efficacy of this novel fabric for the enhancement of a transdermal drug delivery system *in vivo* and *in vitro*.

## Materials and methods

### 

#### Experimental protocols

The clothing used in the present study was constructed using double layered fabric, consisting of an outer layer of polypropylene and an inner layer of nylon with polyurethane. The proportions of each material were 15% polypropylene, 72.5% nylon and 12.5% polyurethane. The fabric also incorporated strategic spatial distribution of hydrophobic and hydrophilic fiber materials. The hydrophobic fiber layer covered the outside of the fabric to prevent water evaporation, whereas the hydrophilic fiber layer was kept in direct contact with the skin to keep it moist after the application of water to the skin (Fig. 1). The control clothing was made of pure organic cotton (100%) and was designed to have a similar fit and shape. All textiles were cut into 15x20 cm squares. To attach the fabric to the four legs of each animal, each piece of fabric was punched at four uniform sites and held together with adhesive tape to prevent clothing removal during the study. All animals were able to maintain a high level of activity while wearing the clothing, but if worn for an entire day, normal activity became difficult to maintain. After applying the topical slimming creams (2 mg/cm^2^) on the abdominal fat of each guinea pig, these procedures were repeated every eight hours per day for 28 days.

#### Animal model

A total of twelve female guinea pigs (six months of age or older; weight, 150-250 g) were purchased from ORIENT BIO, Inc. and used in the current study. All animals were housed individually under controlled environmental conditions (temperature, 18-22˚C; relative air humidity, 30-70%; 15 air changes/h; 12-h light-dark cycle). All procedures involving animals conform to internationally accepted standards and have been reviewed and approved by the Institutional Animal Care and Use Committee of Chung-Ang University, Republic of Korea (IRB number: 2018-9077).

After an acclimation period of 7 days, guinea pigs with a healthy appearance (no abnormal eye movements) were randomly allocated into four groups (n=3) as follows: i) Group 1, untreated control; ii) group 2, topical cosmetic slimming cream alone (Hera Glam Body Slite^®^; Amorepacific Co.); iii) group 3, slimming cream with normal fabric (made of 100% cotton), and iv) group 4, slimming cream with the novel drug delivery fabric (Doctor Slim^®^; Ventex). After all treatments, to take skin samples, guinea pigs were anesthetized using an intramuscular injection of a mixture of ketamine HCL (45 mg/kg of body weight; Ketalar; Yuhan Co., Ltd.) and xylazine (5 mg/kg; Bayer AG). All animals were euthanized using exsanguination immediately after terminal CO_2_ or ketamine HCL-xylazine administration on days 0 and 28.

#### Ultrasound analysis, histological examination and western blot analysis

The changes and reductions in fat layer thickness were calculated with direct contact using by diagnostic ultrasound (Bionet^®^), on day 28, on the abdominal skin. Ultrasound measurements were performed on one representative guinea pig from each group. The amount of fat loss in each guinea pig was evaluated by histological staining with hematoxylin and eosin (H&E; Sigma-Aldrich; Merck KGaA). Tissue biopsy was done after sacrifice on day 0 and day 28. Adipose tissue sections (5 µm thick) were fixed using 4% paraformaldehyde (PFA) for 24 h at room temperature, embedded in paraffin, and transferred to probe-on-plus slides (Thermo Fisher Scientific, Inc.). Deparaffinized skin sections were then stained for 24 h at room temperature using H&E and examined using light microscopy to assess histological changes (magnification, x400). To evaluate the mechanisms underlying the induction of lipid catabolism, western blot analysis was performed on guinea pig skin specimens using antibodies specific for peroxisome proliferator-activated receptor-γ (PPAR-γ) and actin. PPARs are lipid-activated transcription factors that belong to the nuclear hormone receptor family and serve key roles in lipid homeostasis (23). PPAR-γ is part of the PPAR family and is highly expressed in adipose tissue, and serves a crucial role in lipid metabolism, adipocyte function and fat storage (24). Therefore, the expression of PPAR-γ was investigated in adipose tissue following the application of slimming creams, to evaluate the efficacy of drug delivery. Tissue biopsies were collected from abdominal subcutaneous adipose tissue of one representative guinea pig from each group and homogenized in lysis buffer [50 mM Tris-HCl (pH 8.0); 150 mM NaCl; 1 mM EDTA; 1% NP-40 and 0.25% deoxycholate acid] containing a protease inhibitor cocktail (Roche Molecular Diagnostics). The protein concentration of each lysate was quantified using a Bio-Rad DC protein assay kit II (Bio-Rad Laboratories, Inc.). The harvested tissues were homogenized in Pro-prep solution (Intron Biotechnology, Inc.) and lysates were centrifuged at 12,000 x g for 30 min at 4˚C. Total protein (40 µg) was separated by electrophoresis on 10% SDS-polyacrylamide gels. After blocking with 5% non-fat milk, for 2 h at room temperature, blots were probed with antibodies against PPAR-γ (1:1,000; LSBio; cat. no. LS-B651) or actin (1:2,000, LSBio; cat. no. LS-C63547) and incubated for 2 h at room temperature with HRP-conjugated anti-mouse (1:1,000; cat. no. P0447; Dako; Agilent Technologies, Inc.) or anti-rabbit secondary antibodies (1:1,000; cat. no. P0448; Dako; Agilent Technologies, Inc.). Immunoreactive bands were detected using an enhanced chemiluminescence system (GE Healthcare). Protein levels were normalized to those of actin, which was used as a loading control. The transferred proteins were visualized with a Pierce ECL western blotting substrate (Pierce; Thermo Fisher Scientific, Inc.) and quantified by scanning densitometry using Image-Pro Plus 6.0 (Media Cybernetics, Inc.).

#### Fluorescence-based assay of rhodamine B base skin penetration

To visualize the efficiency of the normal and test fabrics on transdermal drug delivery, the topical application of a lipophilic dye, rhodamine B, was performed on the back skin of all group of each the guinea pigs. A confocal laser scanning microscope (Leica SP5 white laser; Leica Microsystems GmbH; magnification, x100) was then used to examine the dye delivery associated with each fabric. Rhodamine B base dye (0.0005 M; Sigma-Aldrich; Merck KGaA) was left to penetrate the skin for 3 h. Immediately after treatment, skin samples were collected and then washed with PBS to remove any residual rhodamine B and embedded in material at the optimal cutting temperature. Fixed skin tissue was frozen by immersion in liquid N_2_-cooled hexane and stored at -80˚C. Transverse sections (60 µm), including the whole right and left ventricles were obtained using a cryostat (Leica CM1325; Leica Microsystems GmbH) and mounted on glass. A DAPI mounting medium kit (OriGene Technologies, Inc.) was used to counterstain the nuclei for 10 min at room temperature, and stained cells were visualized using an Olympus FLUOVIEW FV10i confocal microscope (Olympus Optical Co., Ltd.; magnification x100).

#### In vitro caffeine penetration study

To assess the ability of each fabric to mediate the penetration of a 4% caffeine solution into excised skin samples, the samples were covered with the hydrophobic surface of the normal fabric or the novel drug delivery fabric. Vertically assembled Franz-type diffusion cells were used (5 experiments; 3 cells/experiment). The caffeine release experiment used an FDC-6 transdermal diffusion cell drive console (Logan Instrument Corp.). This system is fitted with a VTC-200 heater circulator and is equipped with a jacketed vertical glass Franz diffusion cell as its main unit. These cells provided a diffusion area of 0.57 cm^2^ and the volume of the receptor compartment was 1 ml. The test formulation (4% caffeine solution; 200 µl/cm^2^) was loaded into the donor compartment before occluding the donor compartments using normal fabric or the test (novel drug delivery) fabric. To maintain sink conditions, PBS (pH 7.4) was used as a receptor. Receptor samples (1 ml) were taken periodically, after which the cells were replenished up to their marked volumes with fresh receptor solution. Receptor solution samples were withdrawn through the receptor sampling port every 2 h. The receptor phase was immediately replenished with an equal volume of fresh receptor phase. Samples were analyzed using high performance liquid chromatography on a Futecs system. The amount of drug released was calculated as a function of time. Each experiment was performed at least five times.

#### Statistical analysis

Statistical comparisons between the treated and untreated groups were performed using one-way ANOVA and a post-hoc Tukey's test (SPSS software version 12.0; SPSS Inc.). Results are expressed as the mean of at least 5 repeats and of at least three independent experiments. ^*^P<0.05 was considered to indicate a statistically significant difference.

## Results

**Figure 2:**
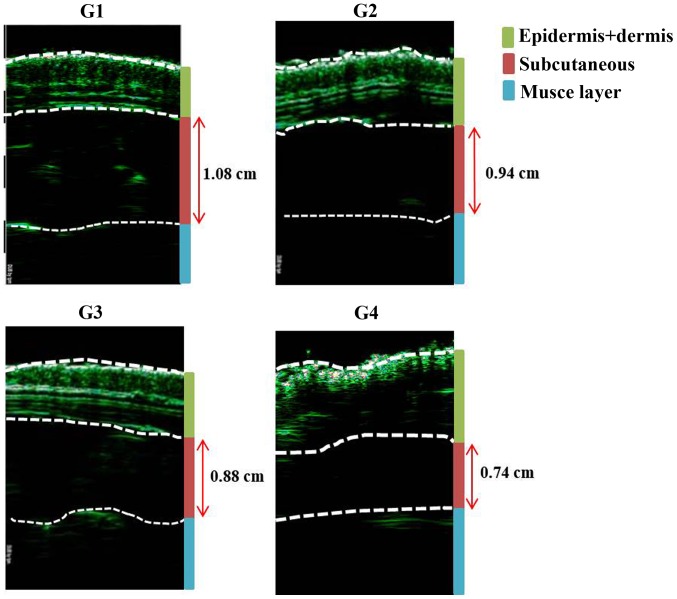
Comparison of fat layer thickness was performed using diagnostic ultrasound assessments on day 28. G1, untreated control; G2, topical cosmetic slimming cream alone without fabric; G3, slimming cream with normal fabric (made of 100% cotton); G4, slimming cream with the novel fabric.

**Figure 3:**
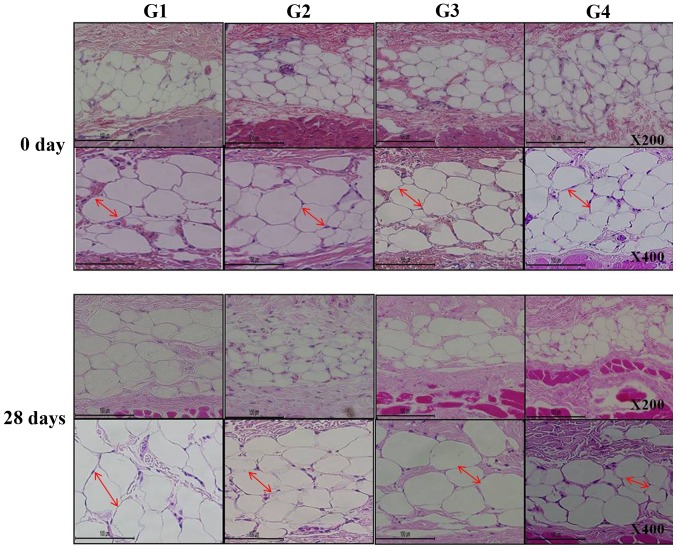
Effects of the novel drug delivery fabric in the application of a slimming cream on adipose tissue. Representative histological images of hematoxylin and eosin staining are presented. At day 0, the adipocytes exhibit normal shapes and sizes. After the experiment, at day 28, adipocyte size appears to be reduced. Red arrows show the adipocytes of each group. Scale bars represent 100 μm. G1, untreated control; G2, topical cosmetic slimming cream alone without fabric; G3, slimming cream with normal fabric (made of 100% cotton); G4, slimming cream with the novel fabric.

**Figure 4:**
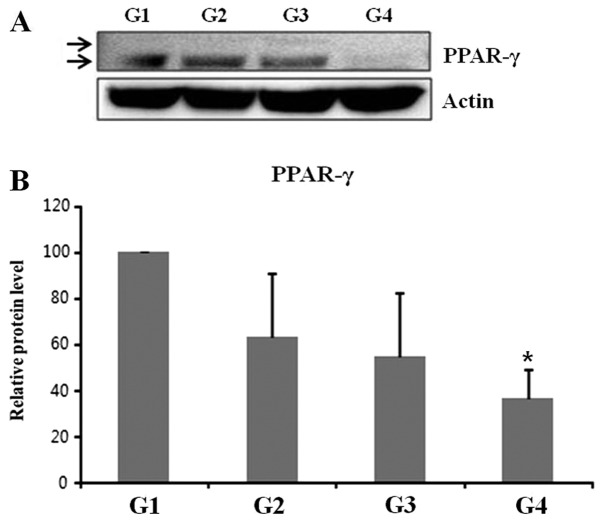
(A) Western blot analysis of adipose tissue lysates to determine the levels of PPAR-γ. (B) The relative amount of PPAR-γ in each group was determined by normalizing its level to actin, which served as a loading control. The amounts of PPAR-γ in each of the treatment groups relative to the control group were subsequently determined. ^*^P<0.05 vs. G1 group. PPAR-γ, peroxisome proliferator-activated receptor-γ; G1, untreated control; G2, topical cosmetic slimming cream alone without fabric; G3, slimming cream with normal fabric (made of 100% cotton); G4, slimming cream with the novel fabric.

**Figure 5:**
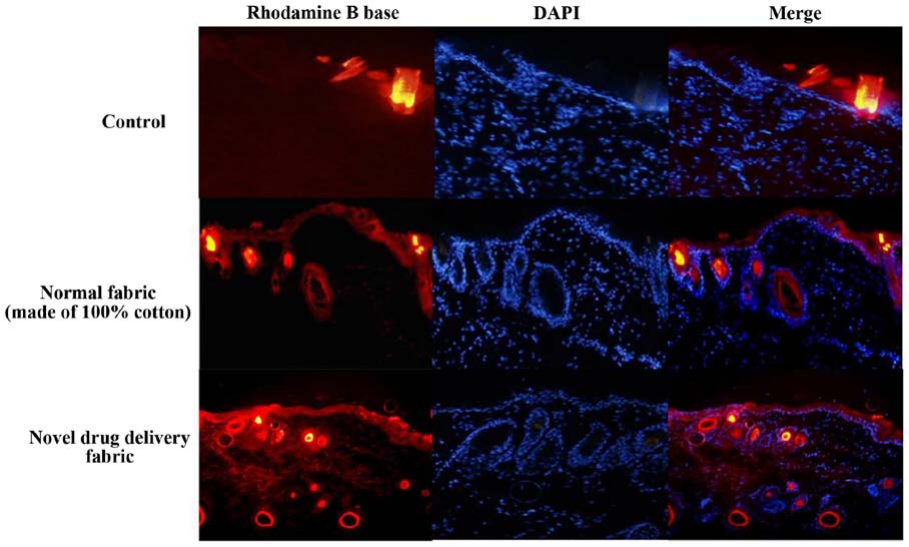
Fluorescence images of rhodamine B base skin penetration at 6 h. Rhodamine B base penetration of guinea pig skin was tested following the application of the dye using normal fabric or the novel drug delivery fabric. Permeated rhodamine B base was detected using fluorescence microscopy. Original magnification, x100.

**Figure 6:**
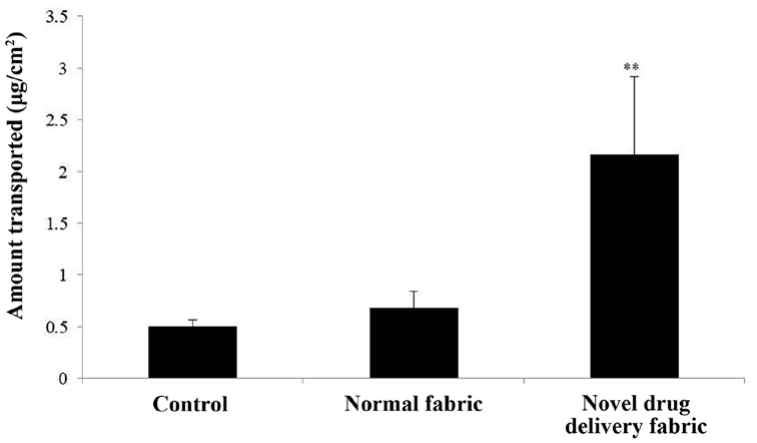
*In vitro* caffeine penetration study using the Franz diffusion cell system. The cumulative amounts of penetrated caffeine skin and fabric over a period of 120 min after the application of a 4% solution are presented. Data are expressed as means ± standard deviations of quintuplicate samples. ^**^P<0.01 vs. control group.

The reduction in thickness of the subcutaneous fat was confirmed using ultrasound examination (thickness of fat layer; group 1, 1.08 cm; group 2, 0.94 cm; group 3, 0.88 cm; group 4, 0.74 cm; Fig. 2). Furthermore, as presented in Fig. 3, histological analysis of the subcutaneous fat indicated that the reduction in fat mass associated with the novel fabric was primarily due to decreased adipocyte size. Additionally, the novel fabric exhibited lower levels of PPAR-γ compared with those from control guinea pigs (Fig. 4). In the fluorescence-based assay of rhodamine B base skin penetration, the novel drug delivery fabric was indicated to enhance the passage of rhodamine B through the guinea pig skin (Fig. 5). For animals in the control group with normal fabric, weak rhodamine B fluorescence was observed in the upper epidermis. In contrast, animals in the novel drug delivery fabric group exhibited strong fluorescence in the subcutaneous and deeper dermis layer (Fig. 5). Furthermore, in the *in vitro* caffeine penetration study, the quantities of caffeine that had permeated the excised skin samples 120 min after application to the normal fabric or the novel drug delivery fabric, are presented in Fig. 6. The novel drug delivery fabric enabled the permeation of 3.18-fold (2.16 µg/cm^2^) more caffeine compared with normal fabric (0.68 µg/cm^2^).

## Discussion

Although a number of methods have been previously evaluated as transdermal drug delivery enhancers, these methods are associated with a number of drawbacks, including low efficacy, limitations regarding molecular weight (<500 Da), high lipophilicity and skin irritation (25,26). Consequently, overcoming the skin barrier in a safe and effective way remains to be a difficulty in the development of novel transdermal drug delivery systems. An aqueous pore pathway has been proposed to mediate the diffusion of active therapeutic agents across the skin under high stress conditions, including excessive hydration (15). This class of permeation enhancers has been demonstrated to increase skin permeability by disordering or ‘fluidizing’ the lipid structure of the SC and forming micro cavities within the lipid bilayers, which increases the diffusion coefficient of a drug (16,17). Excessive hydration utilizes the preexisting scattered lacunae that are embedded in the lipid bilayer. These lacunae expand and form continuous water channels that facilitate the diffusion of hydrophilic and lipophilic permeants (27). Therefore, occlusion can increase absorption, especially for hydrophilic compounds (28). Furthermore, a previous study assessed the effect of clothing on physiological parameters, including skin hydration (17). Occlusion, which can be produced by fabric that maintains hydrophilic conditions for the skin, partially hinders the loss of skin humidity by increasing the water content of the horny layer (29).

Based on these observations, a novel fabric was developed for transdermal drug delivery and an *in vivo* and *in vitro* study was performed to assess its efficiency. The results indicated that components of the slimming cream exhibited increasing permeation into deeper skin layers when administered with the novel fabric compared with a different fabric.

Although the fabric was not tested on human skin, this fabric's ability to enhance drug penetration may be explained by is the skin hydration effect. Fabric is known to serve an important role in the maintenance of liquid and moisture levels near the skin surface (30). By placing a novel fabric on the surface of the skin, a film can form over the skin surface. This film leads to high levels of hydration in the SC. This hydration causes swelling of the SC and induces openings that enhance the penetration of the given permeant. Therefore, the fabric enables the drug to pass through the lipid bilayers of the SC, consequently increasing its permeation. Another possible explanation is that the skin hydration that is performed by the fabric may also affect skin temperature. Water evaporation normally absorbs heat and helps regulate body temperature in response to environmental changes (30). Furthermore, fabric with hydrophilic properties has been proposed to have physiological significance by reducing heat strain and maintaining skin temperature (31). Therefore, clothing serves an important role in the regulation of body temperature and heat loss. Local increases in skin temperature have also been reported to increase blood flow, which in turn increase the rate of permeation or transport of active substances into the skin (26). A previous study concluded that the application of heat (42-44˚C) for 4 h was sufficient to decrease the time required for the patch to deliver steady-state serum concentrations of fentanyl, from 14-18 to 3-4 h (26). Although the skin temperature was not measured in the current study, this explanation is one possible mechanism by which the novel fabric was observed to enhance drug delivery.

The small sample size of the current study limited the statistical power to detect significant differences. Skin hydration levels were also not measured using a corneometer and the amount of transepidermal water loss was also not determined, which would have aided in clarifying the effect of the novel fabric on skin hydration. Additionally, as aforementioned, if skin hydration can contribute to the enhanced transcutaneous penetration, an additional control experiment using a film or membrane occlusive environment is required. The measurement of PPAR-γ, which was conducted in the current study, is limited in explaining the mechanism. However, the antibodies commercially available that are reactive against guinea pig adipose tissue are limited. Therefore, experiments using guinea pigs usually utilize the measurement of PPAR-γ to analyze changes in fat and adipocyte layer. In future studies, further tests that are based on a variety of results should be conducted in the future. Finally, the novel fabric had yet to be tested on human skin. This limitation could be overcome in the future after producing sufficient amounts of fabric and conducting large-scale laboratory experiments or clinical trials on humans. Nevertheless, compared with transdermal delivery systems, the use of this novel fabric may exhibit advantages: i) It increases patient compliance by providing a simple route of administration, and ii) it minimizes the risk of trauma or any other tissue injury. Therefore, enhancing delivery of bioactive molecules through the skin with fabrics including the novel fabric characterized in the present study, promises to create exciting new opportunities in the development of novel and improved techniques for drug delivery through the skin.

In conclusion, the current study demonstrated that a novel fabric for a transdermal drug delivery system enhances penetration of molecules through the skin. Additional studies investigating the potential of using drug delivery fabrics to administer drugs are required to support the findings of the present study.

## Data Availability

The datasets used and/or analyzed during the current study are available from the corresponding author on reasonable request.
